# Structural semiconductor-to-semimetal phase transition in two-dimensional materials induced by electrostatic gating

**DOI:** 10.1038/ncomms10671

**Published:** 2016-02-12

**Authors:** Yao Li, Karel-Alexander N. Duerloo, Kerry Wauson, Evan J. Reed

**Affiliations:** 1 Department of Applied Physics, Stanford University, Stanford, California 94305, USA; 2 Department of Material Science and Engineering, Stanford University, Stanford, California 94305, USA; 3 Klipsch School of Electrical and Computer Engineering, New Mexico State University, Las Cruces, New Mexico 88003, USA

## Abstract

Dynamic control of conductivity and optical properties via atomic structure changes is of technological importance in information storage. Energy consumption considerations provide a driving force towards employing thin materials in devices. Monolayer transition metal dichalcogenides are nearly atomically thin materials that can exist in multiple crystal structures, each with distinct electrical properties. By developing new density functional-based methods, we discover that electrostatic gating device configurations have the potential to drive structural semiconductor-to-semimetal phase transitions in some monolayer transition metal dichalcogenides. Here we show that the semiconductor-to-semimetal phase transition in monolayer MoTe_2_ can be driven by a gate voltage of several volts with appropriate choice of dielectric. We find that the transition gate voltage can be reduced arbitrarily by alloying, for example, for Mo_*x*_W_1−*x*_Te_2_ monolayers. Our findings identify a new physical mechanism, not existing in bulk materials, to dynamically control structural phase transitions in two-dimensional materials, enabling potential applications in phase-change electronic devices.

## Abstract (web summary)

Control of conductivity and optical properties via atomic structure changes is of technological importance in information storage. Here, Li *et al.* show that electrostatic gating has the potential to drive structural semiconductor-to-semimetal phase transitions in some monolayer transition metal dichalcogenides.

## Graphical abstract

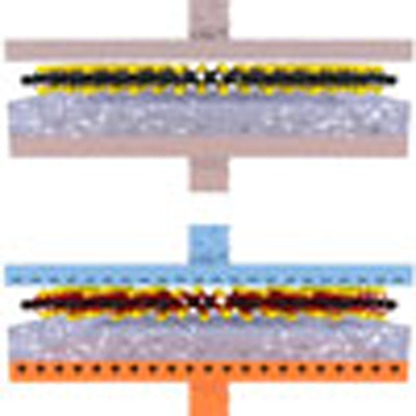

Structural phase transitions yielding a change of electrical conductivity are a topic of long-standing interest and importance^1^,^2^. Two of the most studied phase-change material classes for electronic and optical applications are metal oxide materials^3^,^4^ and GeSbTe alloys^5^, both having a large electrical contrast. For example, the metal oxide material vanadium dioxide (VO_2_) is reported to exhibit a structural metal–insulator transition near room temperature at ultrafast timescales, which can be triggered by various stimuli including heating^6^, optical^7^ excitations and strain^8^. GeSbTe alloys can undergo reversible switching between amorphous and crystalline states with different electrical resistivity and optical properties. This is usually achieved by Joule heating employed in phase-change memory applications^9^,^10^. These materials are distinguished from the myriad materials that exhibit atomic structural changes by the proximity of a phase boundary to ambient conditions.

Another group of materials that can undergo phase transitions are layered transition metal dichalcogenides (TMDs), which have received recent attention as single- and few-layer materials, although research on bulk TMDs dates back decades^11^,^12^. Early attention has been focused primarily on electronic transitions between incommensurate and commensurate charge density wave^13^,^14^ phases and superconducting phases^15^. Some TMDs have been found to exist in multiple crystal structures^16^, and transitions between them have been demonstrated in group V TMDs (TaSe_2_ and TaS_2_) utilizing an scanning tunnelling microscope (STM) tip^17^,^18^. These reported transitions in TaSe_2_ and TaS_2_ are between two metallic phases. Recently, group VI TMDs have attracted increasing attention because they can exist in a semiconducting phase^19^. Recent computational work indicates that structural transitions between phases of large electrical contrast in some exfoliated two-dimensional (2D) group VI TMDs can be driven by mechanical strain^20^. Excess charges transferred from chemical surroundings are also reported to induce structural phase transitions in 2D group VI TMDs^21^,^22^,^23^,^24^. One would like to know the threshold charge density required to induce these transitions and whether these transitions could be dynamically controlled by electrostatic gating, utilizing standard electronic devices.

Here we show the potential of phase control in some monolayer TMDs using electrostatic gating device configurations. In this work, we use density functional theory (DFT) to determine the phase boundaries of single-layer MoS_2_, MoTe_2_, TaSe_2_ and the alloy Mo_*x*_W_1−*x*_Te_2_. We consider MoS_2_ because it has received considerable attention as an exceptionally stable semiconductor, and MoTe_2_ because DFT calculations indicate that its energy difference between semiconducting and semimetallic phases is exceptionally small among Mo- and W-TMDs^20^. We calculate the phase boundaries at conditions of constant charge and constant voltage, the electrical analogues to mechanical conditions of constant volume and constant pressure, respectively. We find that a surface charge density of less than −0.04 *e* or greater than 0.09 *e* per formula unit is required to observe the semiconductor-to-semimetal phase transition in undoped monolayer MoTe_2_ under constant-stress conditions (*e* is the elementary electric charge) and a much larger value of approximately −0.29 *e* or 0.35 *e* per formula unit is required in the undoped monolayer MoS_2_ case. The charge densities discussed in this work refer to excess charge density and should not be misinterpreted as the electron or hole density in a charge-neutral material that one might obtain from chemical doping. We also study the potential of phase control in monolayer MoTe_2_ and TaSe_2_ through electrostatic gating using a capacitor structure. We discover that a gate voltage as small as a few volts for some choices of gate dielectric can be applied to drive the phase transition in monolayer MoTe_2_ using a capacitor structure. While the required field magnitudes are large and may be challenging to achieve, we find that the transition gate voltage may be reduced to 0.3–1 V and potentially lower by substituting a specific fraction of W atoms within MoTe_2_ monolayers to yield the alloy Mo_*x*_W_1−*x*_Te_2_. To accomplish these calculations, we have developed a DFT-based model of the electrostatically gated structure (Supplementary Figs 1 and 2; Supplementary Note 1). This approach is validated by comparing to direct DFT simulations in Supplementary Fig. 3 and Supplementary Note 2.

## Results

### Crystal structures

**Figure 1:**
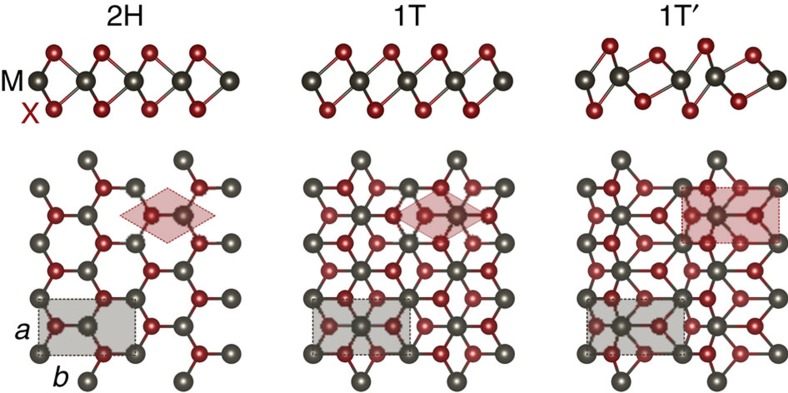
Three crystal structures of monolayer TMDs. The top schematics show cross-sectional views and the bottom schematics show basal plane views. The grey atoms are transition metal atoms and the red atoms are chalcogen atoms; in all three phases, a layer of transition metal atoms (M) is sandwiched between two chalcogenide layers (X). The semiconducting 2H phase has trigonal prismatic structure, and the metallic 1T and semimetallic 1T′ phases have octahedral and distorted octahedral structures, respectively. The grey shadow represents a rectangular computational cell with dimensions *a* × *b*, and the red shadow represents the primitive cell.

TMDs are a class of layered materials with the formula MX_2_, where M is a transition metal atom and X is a chalcogen atom. Each monolayer is composed of a metal layer sandwiched between two chalcogenide layers, forming a X–M–X structure^16^ that is three atoms thick. The weak interlayer attraction of TMDs allows exfoliation of these stable three-atom-thick layers. Given the crystal structures reported in the bulk, we expect that exfoliated monolayer TMDs have the potential to exist in the crystal structures shown in Fig. 1. Figure 1 shows the X atoms with trigonal prismatic coordination, octahedral coordination or a distorted octahedral coordination around the M atoms^16^,^20^,^25^,^26^. We will refer to these three structures of the monolayer as the 2H phase, 1T phase and 1T′ phase, respectively. Symmetry breaking in the 1T′ leads to a rectangular primitive unit cell.

Among these 2D TMDs, the Mo- and W-based materials have attracted the most attention because their 2H crystal structures are semiconductors with photon absorption gaps in the 1–2 eV (ref. 27) range, showing potential for applications in ultrathin flexible and nearly transparent 2D electronics. Radisavljevic *et al.*^28^ fabricated single-layer MoS_2_ transistors of high mobility, large current on/off ratios and low standby power dissipation. Unlike group IV and group V TMDs (for example, TaSe_2_ and TaS_2_), which have been observed in the metallic 1T crystal structure^16^, DFT calculations on the group VI TMDs (Mo and W based) freestanding monolayers indicate that the 1T structure is unstable in the absence of external stabilizing influences^20^. However, group VI TMDs do have a stable octahedrally coordinated structure of large electrical conductivity, which is a distorted version of the 1T phase and referred to as 1T′ structure (Fig. 1). On the basis of DFT calculation results, Kohn–Sham states of this 1T′ crystal structure have metallic or semimetallic characteristics, consistent with previous experiments^16^. This octahedral-like 1T′ crystal structure has been observed in WTe_2_ under ambient conditions^16^,^29^, in MoTe_2_ at high temperature^29^ and in lithium-intercalated MoS_2_ (ref. 25). There is recent experimental evidence that few layer films of the T′ phase of MoTe_2_ exhibit a bandgap that varies from 60 meV to zero with variations in number of layers^30^.

The relative energies of Mo- and W-based TMDs monolayer crystals shown in Fig. 1 have been calculated using semilocal DFT with spin–orbit coupling, shown in Supplementary Fig. 4. These results are consistent with experimental evidence that the bulk form of WTe_2_ is stable in the metallic 1T′ phase, while other Mo- and W-dichalcogenides are stable in the semiconducting 2H phase^16^. These calculations indicate that the switch from semiconducting 2H phase to semimetallic 1T′ phase in monolayer MoTe_2_ requires the least energy (31 meV per formula unit), suggesting the potential for a transition that is exceptionally close to ambient conditions. Therefore, we choose to focus on determining the phase boundary of monolayer MoTe_2_. While the computed energy difference between 2H and 1T′ is considerably larger for MoS_2_ (548 meV per formula unit), we also compute phase boundaries for this monolayer at constant charge because it has received more attention in the laboratory to date. Among 2D group VI TMDs, monolayer MoS_2_ has attracted the most experimental attention for its stability and relative ease of exfoliation and synthesis. Monolayer MoTe_2_ has also been exfoliated^31^,^32^ and its synthesis is a fast-developing field.

### Energy calculations for charged monolayers

**Figure 2:**
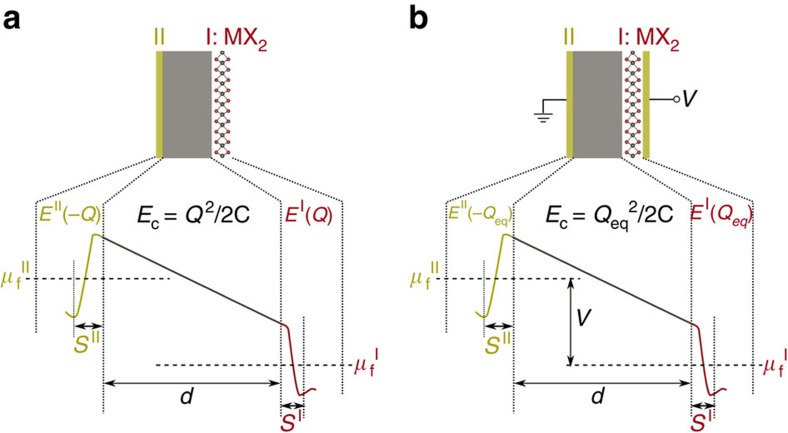
Energy calculations for systems containing a charged monolayer. Layer I is a monolayer TMDs with a Fermi level *μ*_f_^I^, and plate II has a Fermi level of *μ*_f_^II^. Charge *Q* on the monolayer TMDs is fixed in **a**, whereas the voltage *V* is fixed in **b** giving rise to an equilibrium charge *Q*_eq_. A dielectric medium of thickness *d* and capacitance *C*, which can be vacuum, is sandwiched between the monolayer and the plate. Distance *s*^I^ is the separation between the centre of the monolayer and the right surface of the dielectric medium, and *s*^II^ is the separation between the surface atoms of plate II and left surface of the dielectric medium. The total energy in the fixed charge case is the sum of three parts: energy stored in the dielectric medium *E*_c_, energy of the plate II *E*^II^, and energy of the charged monolayer monolayer TMDs *E*^I^.

We examine two distinct thermodynamic constraints for a system containing a charged monolayer. In one scenario, the monolayer is constrained to be at constant excess charge, as shown in Fig. 2a; in the other, the monolayer is constrained to be at constant voltage, as shown in Fig. 2b. These are the electrical analogues to mechanical conditions of constant volume and constant pressure, respectively. The electrical contact depictions in Fig. 2b and subsequent figures are schematic and could be accomplished in other manners, for example, side contacts. In Fig. 2b, the charge is assumed to be stored in the monolayer rather than the metal contact. Layer I is a monolayer TMDs with a Fermi level 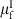, and plate II has a Fermi level of 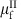. A dielectric medium of thickness *d* and capacitance *C* is sandwiched between monolayer TMDs and plate II. This dielectric medium can be vacuum. Distance *s*^I^ is the separation between the centre of monolayer TMDs I and the right surface of the dielectric medium, while *s*^II^ is the separation between the surface atoms of plate II and the left surface of the dielectric medium. (See Supplementary Figs 5–7, Supplementary Table 1 and Supplementary Note 3 for more details about distance parameters.)

When the charge *Q* on the monolayer is fixed, the total energy of the system *E*(*Q*) is the sum of three parts: energy stored in the dielectric medium (*E*_c_), energy of the plate II (*E*^II^) and energy of the charged monolayer TMDs (*E*^I^), as shown in Fig. 2.


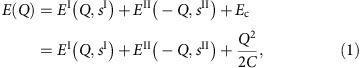


where *C* is the capacitance of the dielectric medium. *E*^I^(*Q*=0, *s*^I^) is the ground-state energy of the electrically neutral monolayer TMDs and *E*^I^(*Q*, *s*^I^)−*E*^I^(*Q*=0, *s*^I^) is the energy required to move electrons *Q* from the Fermi level of the monolayer TMDs to the dielectric surface. *E*^II^(−*Q*, *s*^II^) is defined analogously. We take the monolayers to be undoped in this work.

The first term in equation (1), *E*^I^(*Q*, *s*^I^), is calculated using DFT for each phase of the monolayer TMDs to yield a *E*(*Q*) for each monolayer phase (see Supplementary Figs 1 and 2, and Supplementary Note 1 for calculation details). The phase change does not enter into the third term in equation (1) or change the capacitance of the dielectric medium *C*.

We take plate II to be a bulk metal with a work function *W* so that the second term in equation (1) can be approximately written as:


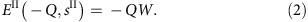


When the voltage is fixed rather than the charge, the grand potential Φ_G_(*Q*, *V*) becomes the relevant thermodynamic energy defined as:


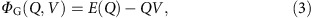


where *E*(*Q*) is computed using equation (1). The *QV* term in this expression represents external energy supplied to the system when the charge *Q* flows through an externally applied voltage *V*. The equilibrium charge *Q*_eq_ can be calculated through minimization of the grand potential at a given gate voltage *V*.


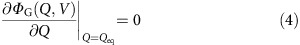


Applying the computed *Q*_eq_(*V*) to equation (3), we can obtain the equilibrium grand potential as a function of gate voltage 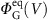.


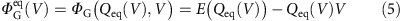


Hereafter, we omit the superscript ‘eq' for the equilibrium grand potential 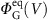.

In addition to the electrical constraint, the nature of the mechanical constraint on the monolayer is also expected to play a role in the phase boundary, discussed in Supplementary Note 4.

### Phase boundary at constant charge

The distinction between the constant charge and voltage cases is most important when a phase transformation occurs. We discover that the transition between semiconducting 2H-TMDs and semimetallic 1T′-TMDs can be driven by excess electric charge (positive or negative) in the monolayer. A constant charge condition exists when the charge on the monolayer remains constant during the phase transition as if it is electrically isolated. An approximate condition of constant charge could exist when adsorbed atoms or molecules donate charge to the monolayer.

**Figure 3:**
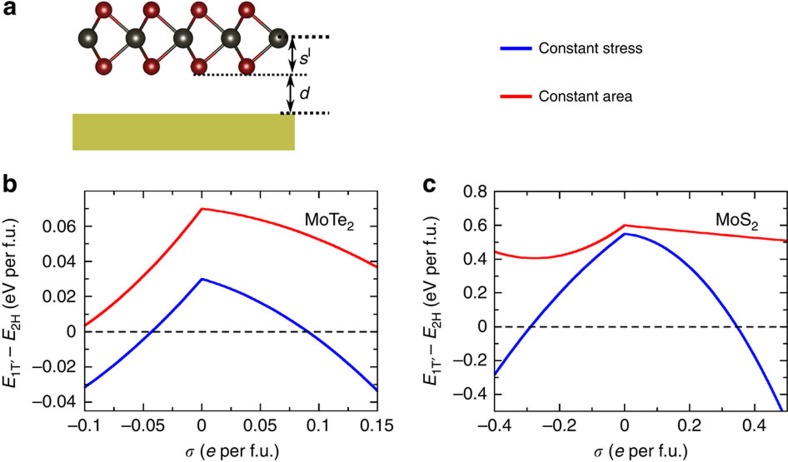
Phase boundary at constant charge in monolayer MoTe_2_ and MoS_2_. (**a**) Schematic representation of a monolayer TMDs separated by vacuum from an electron reservoir, for example, the surface of a metal. The internal energy difference between 2H and 1T′ phases 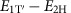 changes with respect to the charge density *σ* as shown in **b** and **c**. The units are per formula unit (f.u.). The blue line represents constant-stress (stress-free) case, in which both 2H and 1T′ are structure relaxed. The red line represents the constant-area case, in which the monolayer is clamped to its 2H lattice constants. (**b**) Semiconducting 2H-MoTe_2_ is a stable phase and semimetallic 1T′-MoTe_2_ is metastable when the monolayer is charge neutral. However, 1T′-MoTe_2_ is more thermodynamically favourable when the monolayer is charged beyond the positive or negative threshold values. The charge thresholds exhibit a significant dependence on the relaxation of lattice constants, indicating that the precise transition point may be sensitive to the presence of a substrate. (**c**) MoS_2_ is stable in the 2H structure when charge neutral. The magnitude of charge required for the transition to 1T′ is larger than for MoTe_2_. In both cases, transition at constant stress is more easily induced than the transition at constant area.

Figure 3 presents the energy difference between the 2H and 1T′ phases as a function of the charge density in the monolayer. Figure 3a is a schematic of the system. The monolayer TMD is a distance *d* away from the electron reservoir (metal electrode). Because the dielectric medium is vacuum in this schematic, *E*^I^ and *E*_c_ in equation (1) can be combined, which can be understood from Fig. 2a, and equation (1) can be rewritten as:


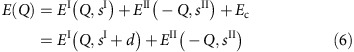


When computing the energy difference between a system where the monolayer is in the 1T′ phase and another system where the monolayer is in the 2H phase 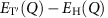, the terms *E*^II^(−*Q*, *s*^II^) in equation (6) cancel, leading to,


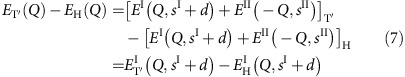


Equation (7) shows that the energy difference depends on *s*^I^+*d* rather than *s*^I^ and *d* independently. Variation of the results of Fig. 3b,c with the separation *s*^I^+*d* (chosen to be 15 Å in Fig. 3) is weak or none as shown in Supplementary Fig. 7.

The blue lines are constant-stress (stress-free) cases, in which both phases exhibit minimum energy lattice constants and atomic positions. This condition is expected to hold when the monolayer is freely suspended or is not constrained by friction on a substrate. The red lines represent constant-area cases, where the monolayer is clamped to its 2H lattice constants. This condition might be expected to hold when there is a strong frictional interaction between the monolayer and substrate preventing the monolayer from relaxing freely.

Figure 3b shows that semiconducting 2H-MoTe_2_ has lower free energy and is the equilibrium state when the monolayer is electrically neutral or minimally charged. For the stress-free case (blue line), when the charge density is between −0.04 *e* and 0.09 *e* per formula unit, 2H-MoTe_2_ is the thermodynamically stable phase. These charge densities correspond to −3.7 × 10^13^ and 8.2 × 10^13^ *e* cm^−2^, respectively. Outside this range, semimetallic 1T′-MoTe_2_ will become the equilibrium phase and a transition from the semiconducting 2H phase to the semimetallic 1T′ phase will occur.

In the constant-area case (red line) in Fig. 3b, a considerably larger charge density is required to drive the phase transition. This suggests that the precise transition point may be sensitive to the presence of a substrate and that the detailed nature of the mechanical constraint of the monolayer may play a substantive role in the magnitude of the phase boundaries. The higher transition charge in this case can be understood by considering that the energy of the strained T′ phase is higher than that of the zero stress T′ phase, pushing the phase boundary to larger charge states.

Figure 3c shows that the transition in monolayer MoS_2_ requires much larger charge density than the MoTe_2_ case. If the negative charge density is >0.29 *e* per MoS_2_ formula unit, semimetallic 1T′-MoS_2_ will have lower free energy and be more stable. For negative charge densities <0.29 *e* per formula unit, semiconducting 2H-MoS_2_ will be energetically favourable. This is consistent with previous experimental reports that adsorbed species donating negative charge to monolayer MoS_2_ can trigger a trigonal prismatic to octahedral structure transformation^24^,^33^. MoS_2_ single layer is reported to adopt a distorted octahedral structure when bulk MoS_2_ is first intercalated with lithium to form Li_*x*_MoS_2_ with *x*≈1.0 and then exfoliated by immersion in distilled water^25^. This is qualitatively consistent with our prediction that a negative charge density >0.29 *e* per MoS_2_ may trigger the phase transition from 2H phase to 1T′ phase MoS_2_. See Supplementary Fig. 8 and Supplementary Note 5 for an intuitive discussion of the mechanism for the charge-induced structural phase transition.

### Phase boundary at constant voltage

Another relevant type of electrical constraint is fixed voltage or electron chemical potential. This constraint is most applicable when the monolayer is in an electrostatic gating structure similar to field-effect transistors made using monolayers. Such a device structure enables a dynamical approach to achieve semiconductor/semimetal phase control in monolayer TMDs, suggesting intriguing applications for ultrathin flexible 2D electronic devices including phase-change memory.

**Figure 4:**
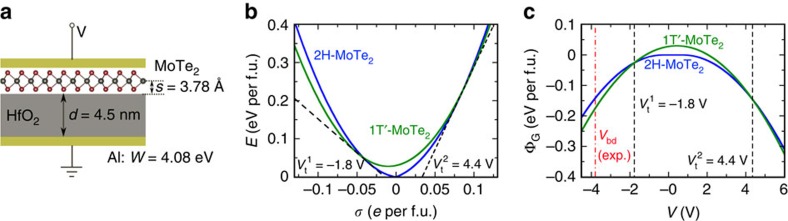
Phase boundary at constant voltage and stress. (**a**) Monolayer MoTe_2_ deposited on top of a HfO_2_ layer of thickness *d*=4.5 nm, which is on top of an aluminum plate of work function *W*=4.08 eV. Voltage *V* is applied between the monolayer and the aluminum plate. (**b**) Plotted is the total energy *E* of the capacitor shown in **a** as a function of the charge density *σ* on monolayer MoTe_2_. (**c**) Plotted is the grand potential Φ_G_ as a function of the gate voltage *V*. The blue line represents a capacitor containing 2H-MoTe_2_, whereas the green line represents a capacitor containing 1T′-MoTe_2_. The two black dashed lines in **b** depict common tangents between the 2H and 1T′ energy surfaces, and in **c** represent intersections of the 2H and 1T′ grand potentials indicating two transition voltages *V*_t_^1^ and *V*_t_^2^. Between the two transition voltages, semiconducting 2H-MoTe_2_ has a lower grand potential and is thermodynamically stable. Outside this range, 1T′ will be more stable. The red dashed line in **c** represents a breakdown voltage^35^ obtained experimentally for a HfO_2_ film of thickness 4.5 nm. The separation between MoTe_2_ centre and the surface of HfO_2_ is assumed to be *s*=3.78 Å, and both 2H and 1T′ are structurally relaxed (constant stress) in **b** and **c**.

Many distinct electrostatic gating device structures can be utilized to realize this dynamic control through a change in carrier density or electron chemical potential of the monolayer. Here we consider a capacitor structure shown in Fig. 4a. A monolayer of MoTe_2_ is deposited on top of a dielectric layer of thickness *d*, which we take to be HfO_2_ with a large dielectric constant of 25 (ref. 34). Monolayer and dielectric are sandwiched between two metal plates between which a voltage *V* is applied. High-dielectric constant material HfO_2_ is chosen to increase the capacitance and hence increase the charge density in the monolayer. The metal plate is chosen to be aluminum with a work function of 4.08 eV. The curves in Fig. 4 assume the monolayer to be at a state of constant stress, with both 2H and 1T′ phases structurally relaxed. We compute the total energy and equilibrium grand potential of this system using equations (1, 2, 3, 4, 5).

Plotted in Fig. 4b is the total energy (equation 1) of the capacitor shown in Fig. 4a as a function of charge density in monolayer MoTe_2_. Two black dashed lines depict common tangents between 2H and 1T′ energy surfaces, the slopes of which are defined by the set of equations,


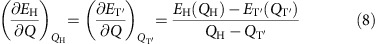


where 
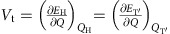
 is the transition gate voltage.

Plotted in Fig. 4c is the equilibrium grand potential (equation 5) as a function of the gate voltage. Two transition voltages are labelled also using black dashed lines. Figure 4b,c shows that a transition gate voltage of −1.6 V or 4.4 V can be applied to drive the phase transition in monolayer MoTe_2_ using the capacitor in Fig. 4a. The experimental breakdown voltage for a 4.5-nm-thick HfO_2_ is reported to be as large as 3.825 V (ref. 35), which is larger than twice the magnitude of the negative transition voltage. This breakdown field in HfO_2_ is larger than some other reports and may depend on the details of growth^36^,^37^. Therefore, employing an appropriate dielectric is likely to be critical here in observing the phase change. Ionic liquids may be employed to help address the challenge of achieving large voltages. Ionic gating has been applied to a variety of TMDs to investigate superconductivity^38^ by measuring I–V curves. However, when a large voltage is applied, it may be challenging to probe structural phase transitions from I–V curves alone due to a large density of charge in the TMDs. Structural characterization approaches, such as Raman spectroscopy, may provide a more direct probe of electrically induced structure phase transitions in monolayer TMDs.

**Figure 5:**
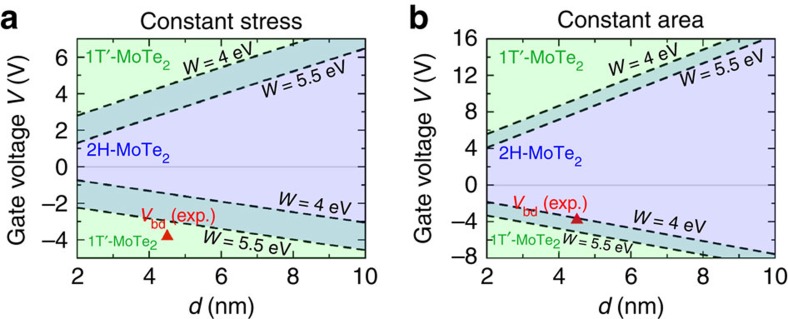
Phase control of MoTe_2_ through gating at constant stress and constant area. (**a**,**b**) Plotted are phase stabilities of monolayer MoTe_2_ with respect to gate voltage *V* and dielectric thickness *d* using the capacitor structure shown in Fig. 4a. In each phase diagram, there exist two phase boundaries that vary with the work function *W* of the capacitor plate. Between the two phase boundaries, semiconducting 2H is more stable, and outside 1T′ is the stable structure. The required transition gate voltage is smaller in the constant-stress case (**a**) than in the constant-area case (**b**). For a constant-stress scenario, a negative gate voltage as small as −1 to −2 V can trigger the semiconducting-to-semimetallic phase transition in monolayer MoTe_2_. The red triangle represents the breakdown voltage of a 4.5-nm-thick HfO_2_ film obtained experimentally^35^.

While the curves in Fig. 4 assume that the monolayer is at a state of zero stress across the transition, Fig. 5 presents calculations for MoTe_2_ at constant stress (Fig. 5a) and constant area (Fig. 5b) utilizing the capacitor structure shown in Fig. 4a. These phase diagrams predict the thermodynamically favoured phase as a function of voltage *V* and thickness *d* of the HfO_2_ dielectric medium. In each phase diagram, there exist two phase boundaries, the positions of which vary with the work function *W* of the capacitor plate. The 2H semiconducting phase of MoTe_2_ is stable between the two phase boundaries, and metallic 1T′-MoTe_2_ is stabilized by application of sufficiently positive or negative gate voltages. The transition voltages increase with the thickness of the dielectric layer. For a capacitor containing a HfO_2_ dielectric layer of thickness <5 nm, a negative gate voltage of approximately −2 V may be applied to drive the semiconductor-to-semimetal phase transition at constant stress (Fig. 5a) but the required voltage increases to approximately −4 V at constant area in Fig. 5b. In analogue with the changes in charge density phase boundaries shown in Fig. 3b, the voltage magnitudes for the transition are larger in constant-area conditions (Fig. 5b) than at constant stress (Fig. 5a). If the substrate constrains the area of the monolayer across the transition through friction, the voltages in Fig. 5b are expected to be applicable. The figure also shows a reported experimental breakdown voltage of a 4.5-nm-thick HfO_2_ film^35^.

Field-effect transistors based on few-layered MoTe_2_ have been reported in ref. 39 using a 270-nm-thick SiO_2_ gate dielectric layer (3.9 dielectric constant) with gate voltages as large as −50 V. For monolayer MoTe_2_ (rather than few layers), our model predicts that a gate voltage >200 V is required to drive the phase transition for this device configuration. Both the increase of dielectric thickness (from 5 to 270 nm) and the decrease of dielectric constant (from 25 for HfO_2_ to 3.9 for SiO_2_ (ref. 35)) will result in larger transition gate voltages than shown in Fig. 5. To observe the 2H-1T′ phase transition in a device, choosing a dielectric medium of large dielectric constant and dielectric performance will be critical.

### Reducing transition gate voltages with the alloy Mo_*x*_W_1−*x*_Te_2_

Monolayer alloys present the possibility for reducing the required gate voltage by varying the chemical composition. Recently, monolayer alloys of Mo- and W-dichalcogenides have attracted increasing attention for their tunable properties^40^,^41^,^42^,^43^,^44^. We hypothesize that the 2H-1T′ transition gate voltage can be tuned to lower values by alloying MoTe_2_-WTe_2_ monolayers. This is because in monolayer MoTe_2_, the 2H phase is energetically favourable by 31 meV per formula unit relative to the 1T′ phase, whereas in monolayer WTe_2_, the energy of the 1T′ phase is 123 meV per formula lower than the 2H phase, as shown in Supplementary Fig. 4. Therefore, one might expect the energy difference between the two charge-neutral phases to be tunable through zero with alloy composition. The smaller the energy difference is, the closer the phase boundary is to ambient condition and the smaller the external force required to drive the phase transition in monolayer TMDs^20^. Therefore, controlling alloy composition is likely to enable tuning of the transition gate voltage.

Earlier experimental reports of the synthesis of the bulk alloy Mo_*x*_W_1−*x*_Te_2_ (ref. 45) and detailed calculations on monolayers^44^ indicate that the phase changes from 2H to 1T′ with increase in W fraction 1−*x*. This indicates that the free energy difference between the 2H and 1T′ phases can be made arbitrarily small by varying *x*, enabling an arbitrary reduction of the gate voltage. However, the precise value of *x* required to achieve a particular transition voltage is likely to depend on a number of factors including synthesis conditions and mechanical constraints^44^,^45^. Here we study an approximate representative atomic configuration for this alloy for *x*=0.67, displayed in Supplementary Fig. 9, with the knowledge that some variation of computed phase diagram can occur with the choice of configuration. Detailed cluster expansion calculations for these monolayer alloys are presented in ref. 44.

**Figure 6:**
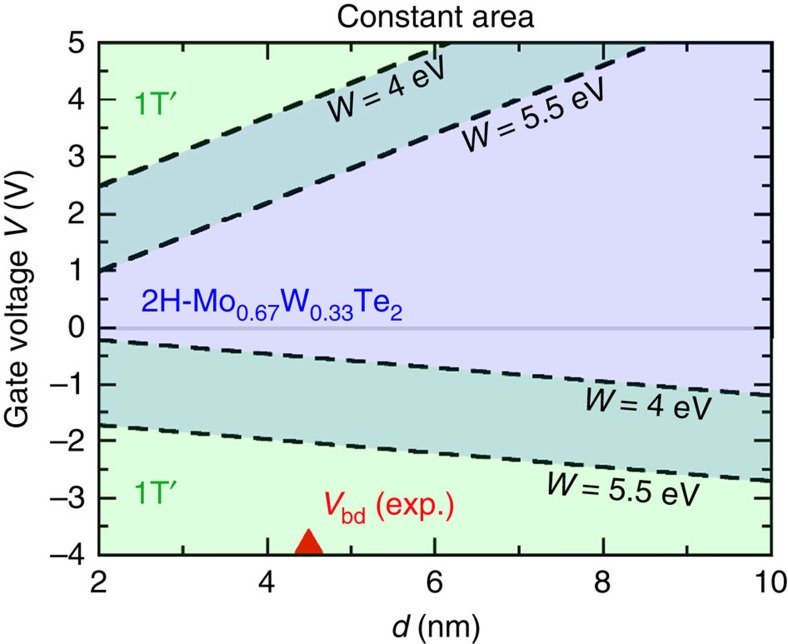
Reducing transition gate voltages with the alloy Mo_*x*_W_1−*x*_Te_2_. Plotted is the phase stability of a representative alloyed monolayer Mo_0.67_W_0.33_Te_2_ with respect to the gate voltage *V* and the dielectric thickness *d* using the capacitor structure as shown in Fig. 4a. The transition is assumed to occur at constant monolayer area. The magnitudes of the transition gate voltages in this alloy are smaller than those of pure MoTe_2_ monolayer indicating the potential for phase boundary engineering.

For the alloy configuration we employed in this work (assumed at constant area), the 2H phase is a semiconductor with a semilocal quasiparticle Kohn–Sham bandgap of ∼0.9 eV, and its free energy is 15 meV lower than the 1T′ phase at constant monolayer area, which is metallic or semimetallic. Figure 6 shows that the 2H-1T′ phase transition in this alloy can be driven by negative gating of a smaller gate voltage than pure MoTe_2_ monolayer. For example, assuming HfO_2_ medium of 4.5-nm thickness and capacitor plate of 4.0 eV work function, the magnitude of negative transition gate voltage can be reduced from 3.6 V (MoTe_2_, constant-area case) to 0.4 V in the constant-area case of Mo_0.67_W_0.33_Te_2_ monolayer.

One might expect that the transition gate voltage in monolayers can be tuned and reduced potentially arbitrarily by controlling the chemical composition of this and other potentially alloys. To enable a structural phase transition driven by a small gate voltage, elements should be selected for alloying so that the energy difference between charge-neutral 2H and 1T′ phases can be tuned through zero with alloy composition. Alternative mechanical constraints placed on the alloy monolayer (for example, constant stress) can also be expected to shift the phase boundary and transition gate voltage.

### Phase transition in Ta-based TMDs

**Figure 7:**
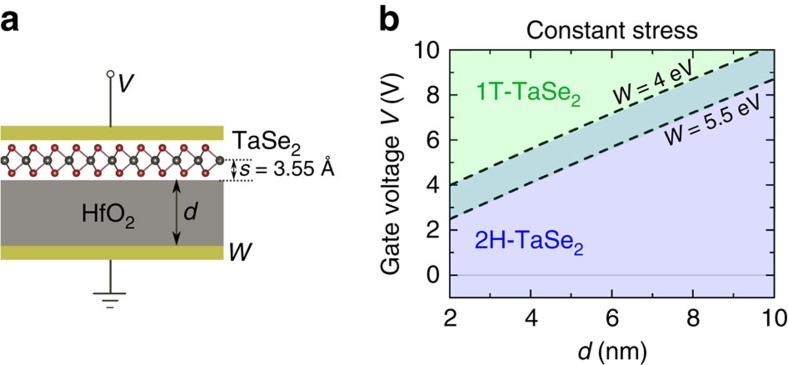
Phase control of monolayer TaSe_2_ through gating at constant stress. (**b**) Plotted is the computed phase diagram of monolayer TaSe_2_ in the capacitor gating structure as shown in **a**. Unlike MoTe_2_, the phase diagram of TaSe_2_ only has one phase boundary, which corresponds to a positive gate voltage. Below the phase boundary, 2H-TaSe_2_ is more stable; above the boundary, 1T has lower energy and is more stable. Intuition for this qualitative difference from MoTe_2_ is provided in Supplementary Note 5.

Electrically induced structural phase changes between 2H and 1T phases in the Ta-based TMDs, TaSe_2_ and TaS_2_, have been reported in experiments using a STM tip^17^,^18^, although the mechanism for this reported effect may differ from the charge-induced effect reported in the present work. As a supplement to the previous calculations on group VI TMDs, we have computed the constant-stress phase diagram of monolayer TaSe_2_ in the capacitor gating structure shown in Fig. 7a. Figure 7b shows that the phase diagram of TaSe_2_ has a phase boundary at only positive gate voltage, qualitatively different from MoTe_2_ and MoS_2_. We find that this difference results from the metallic nature of both 2H- and 1T-TaSe_2_, further discussed in Supplementary Note 5.

A qualitative difference between these calculations and the STM experiment^17^ is the observation of the transition at both signs of STM bias, suggesting that other effects could be at play in the experiment. Further quantitative comparison with the experiment is made challenging by the small separation between the monolayer and the STM tip (Supplementary Fig. 3; Supplementary Note 2).

## Discussion

The electrical dynamical control of structural phase in monolayer TMDs has exciting potential applications in ultrathin flexible 2D electronic devices. If the kinetics of the transformation are suitable, nonvolatile phase-change memory^9^ may be an application. One might expect 2D materials to have energy consumption advantages over bulk materials due to their small thickness. If the kinetics is sufficiently fast, another potential application may be subthreshold swing reduction in field-effect transistors to overcome the scaling limit of conventional transistors^4^. In addition, the change in the transmittance of light due to the phase transition of monolayer TMDs may be employed in infrared optical switching devices, such as infrared optical shutters and modulators for cameras, window coating and infrared antennas with tunable resonance.

To summarize, we have identified a new mechanism, electrostatic gating, to induce a structural semiconductor-to-semimetal phase transition in monolayer TMDs. We have computed phase boundaries for monolayer MoTe_2_, MoS_2_ and TaSe_2_. We discover that changing carrier density or electron chemical potential in the monolayer can induce a semiconductor-to-semimetal phase transition in monolayer TMDs. We find that a surface charge density less than −0.04 *e* or greater than 0.09 *e* per formula unit is required to observe the semiconductor-to-semimetal phase transition in monolayer MoTe_2_ under constant-stress conditions, and a significantly larger value of approximately −0.29 *e* or 0.35 *e* per formula unit is required in the monolayer MoS_2_ case. A capacitor structure can be employed to dynamically control the semiconductor-to-semimetal phase transition in monolayer MoTe_2_ with a gate voltage ∼2–4 V for MoTe_2_. These transition charges and voltages are expected to vary considerably with the nature of the mechanical constraint of the monolayer and also potentially the presence of dopants or Fermi level pinning. While the gate voltages required to observe the transition in MoTe_2_ are likely near breakdown and could be challenging to realize in the lab, we find that the voltage magnitudes can be reduced arbitrarily by alloying Mo atoms with substitutional W atoms to create the alloy Mo_*x*_W_1−*x*_Te_2_.

## Methods

### Electronic structure calculations

All periodic DFT calculations were performed within the Vienna Ab Initio Simulation Package^46^, version 5.3.3, using the projector augmented-wave^47^ method and the plane-wave basis set with a kinetic energy cutoff of 350 eV. Electron exchange and correlation effects were treated using the Generalized Gradient Approximation (GGA) functional of Perdew, Burke and Ernzerhof^48^. An 18 × 18 × 1 Monkhorst-Pack^49^ k-point mesh was utilized to sample the Brillouin zone. The convergence thresholds for electronic and ionic relaxations were chosen to be 0.5 × 10^−8^ eV per MX_2_ formula unit and 0.5 × 10^−7^ eV per MX_2_ formula unit, respectively. A Gaussian smearing of 50 meV was used. The computational cell length is 36 Å along the *c* axis. Spin–orbit coupling is employed in all DFT calculations. The ionic relaxations were performed using conjugate gradient algorithm.

All calculations in this work were performed at zero ionic temperature, omitting the vibrational component of the free energy. 20 has shown that inclusion of vibrational free energy and temperature would shift the phase boundaries closer to ambient conditions and lower the energy required to switch the phases. Therefore, one would expect inclusion of these effects to decrease the magnitude of the transition charge density and gate voltage calculated in this work. Also, the change of bandgap width is expected to affect 2H-1T′ phase boundary, as further discussed in Supplementary Fig. 10 and Supplementary Note 6. See Supplementary Note 7 for a discussion on vacuum electronic states.

## Additional information

**How to cite this article:** Li, Y. *et al.* Structural semiconductor-to-semimetal phase transition in two-dimensional materials induced by electrostatic gating. *Nat. Commun.* 7:10671 doi: 10.1038/ncomms10671 (2016).

## Supplementary Material

Supplementary Information Supplementary Figures 1-10, Supplementary Table 1, Supplementary Notes 1-7 and Supplementary References.
