# Supplementary material for: Structural semiconductor-to-semimetal phase transition in two-dimensional materials induced by electrostatic gating
Source: Nat Commun. 2016 Feb 12;7:10671. doi: 10.1038/ncomms10671 (PMC4754345; doi:10.1038/ncomms10671)
Supplement: Supplementary Information — Supplementary Figures 1-10, Supplementary Table 1, Supplementary Notes 1-7 and Supplementary References. [file ncomms10671-s1.pdf]

Supplementary Information

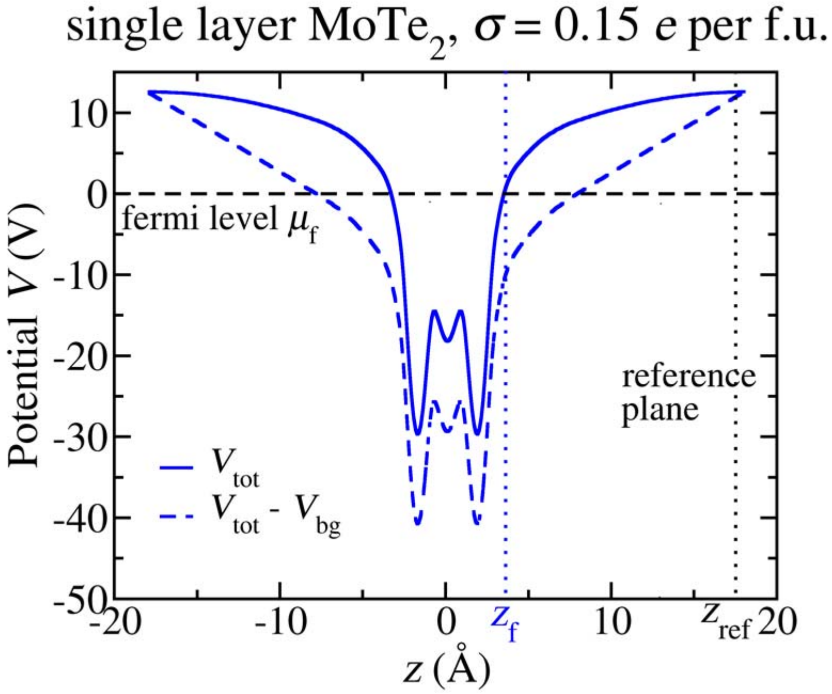

Supplementary Figure 1: Electronic Kohn-Sham potential profile of a charged monolayer $MoTe_2$ calculated using PBE-DFT. Plotted is the averaged electronic Kohn-Sham potential along the vacuum direction *z* of a charged monolayer 2H-$MoTe_2$, as calculated using DFT. The monolayer is positively charged by 0.15 *e* per formula unit. The blue solid line shows the total potential V_{tot}, including the potential of the charged monolayer and of the uniform compensating background charge V_{bg}. The blue dashed line shows the potential of the charged monolayer only, i.e. $V_{tot} – V_{bg}$. The black dashed line shows the Fermi level μ_f as calculated using DFT. The Fermi reference position (taken to be the intersection between the Fermi level and the total potential) is a distance z_f away from the center of the monolayer. A reference plane is positioned at a distrance z_{ref} away from the monolayer center.

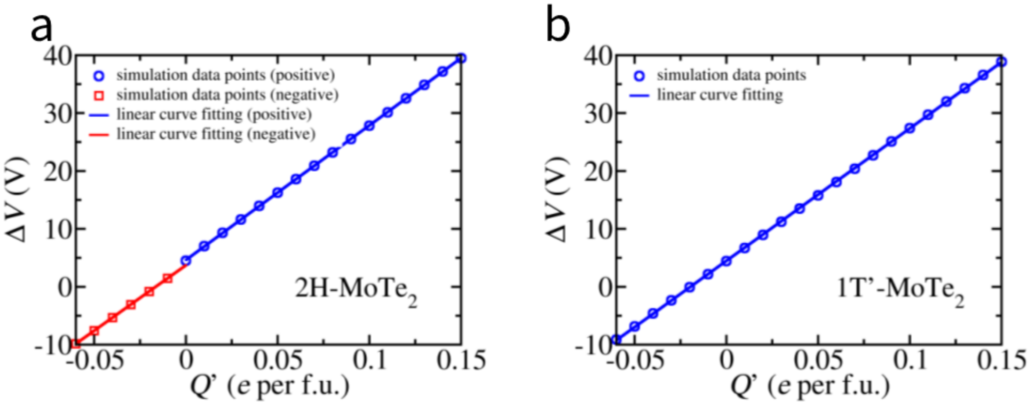

Supplementary Figure 2: Curve fitting of Equation (5) in Supplementary Note 1 for monolayer 2H-$MoTe_2$ (a) and 1T'-$MoTe_2$ (b). Circles and squares represent DFT-simulation data points and curves represent linear curve fitting results. The discontinuity at Q' = 0 in panel (**a**) is due to the band gap in semiconducting 2H-$MoTe_2$.

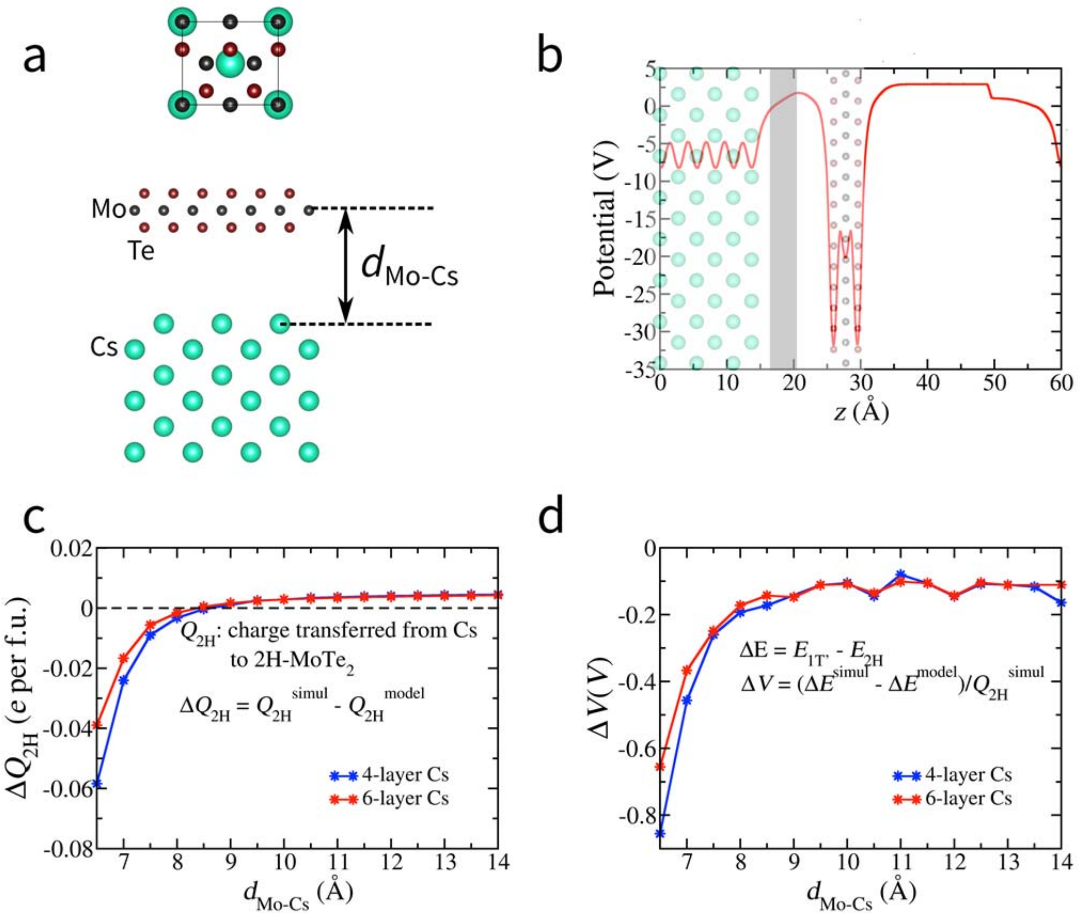

Supplementary Figure 3: Test case of a monolayer $MoTe_2$ on top of a Cs substrate. A system consisting of a monolayer $MoTe_2$ on top of a Cs substrate, as shown in panel **a**, is simulated using DFT and computed using the model described in Supplementary Note 2. The distance between the center of the monolayer and the substrate surface atom centers is $d_{\text{Mo-Cs}}$. The simulation and model prediction results are compared in panels **c** and **d**. Plotted in panel **b** is the averaged electronic Kohn-Sham potential of the system along the vacuum direction *z*, as calculated using DFT. Plotted in **c** is the difference between the simulation and model prediction for the charge transferred from the substrate to 2H-$MoTe_2$ as a function of $d_{\text{Mo-Cs}}$. Plotted in panel **d** is the voltage difference

between the simulation and model prediction, estimated by dividing the difference between the simulation and model prediction for the energy difference of 2H and 1T' phases by the excess charge transferred to 2H-$MoTe_2$. In panels **c** and **d**, the Cs substrate consists of four layers for the red curves and six layers for the blue curves.

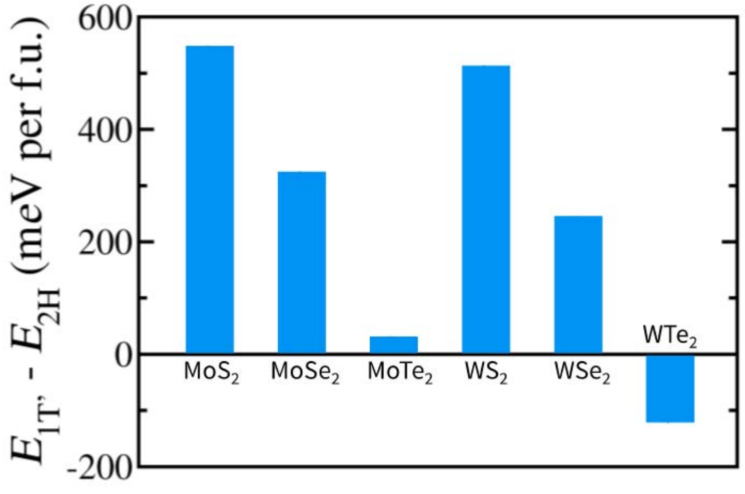

Supplementary Figure 4: Phase energetics of 2H and 1T' monolayers. Plotted are semilocal DFT-calculated energy differences per formula unit between freely suspended charge-neutral 2H and 1T' phases of monolayer Mo- and W-dichalcogenides, with spin-orbit coupling effects included. Previous calculations have shown that the smaller the energy difference is, the closer the phase boundary is to ambient conditions and the smaller the external force required to drive the phase transition in monolayer TMDs[1]. Therefore, monolayer TMDs with small energy difference between charge-neutral 2H and 1T' phases should be selected to display the phase transition driven by electrostatic gating.

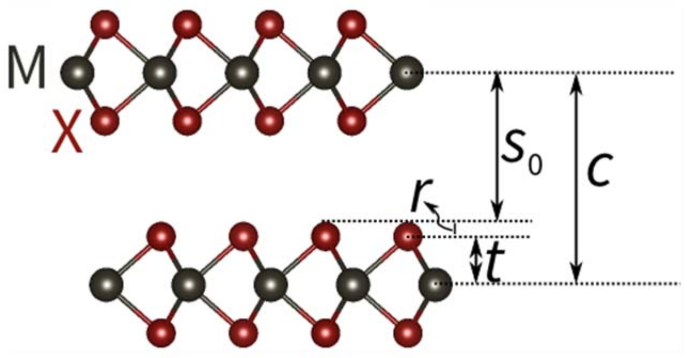

Supplementary Figure 5: Crystal structure and distance parameters of 2H phase bulk TMDs. Grey spheres (M) represent transition metal atoms, and red spheres (X) represent chalcogenide atoms.

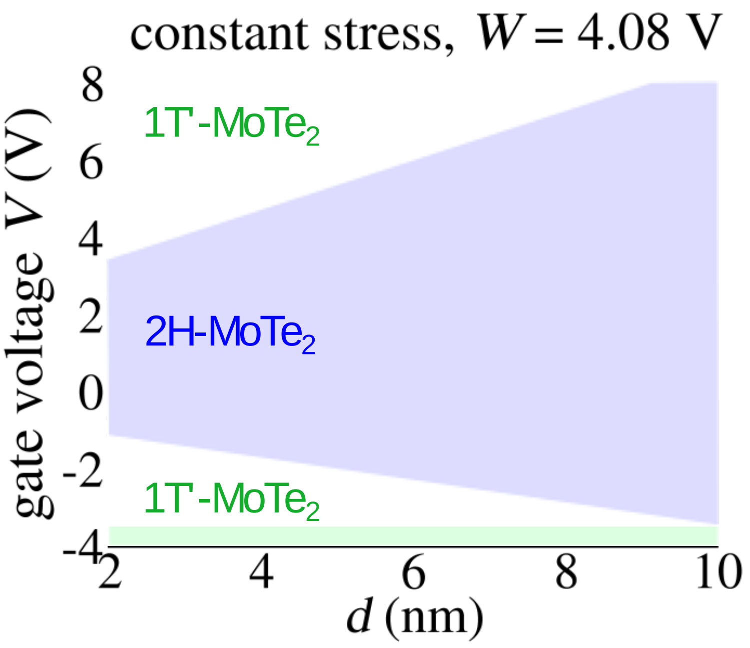

Supplementary Figure 6: Dependence of transition gate voltages on distance parameter s. Plotted is the phase stability of monolayer $MoTe_2$ with respect to the gate voltage V and the dielectric thickness d using the capacitor structure as shown in Fig. 4(a) of main text. The transition is assumed to occur at constant stress. The metal plate is chosen to be aluminum of work function W = 4.08 eV. The phase boundaries vary in position with the distance s between $MoTe_2$ center and the surface of dielectric medium by 0.4 V for negative charging and 1.0 V for positive charging over a physical range of s.

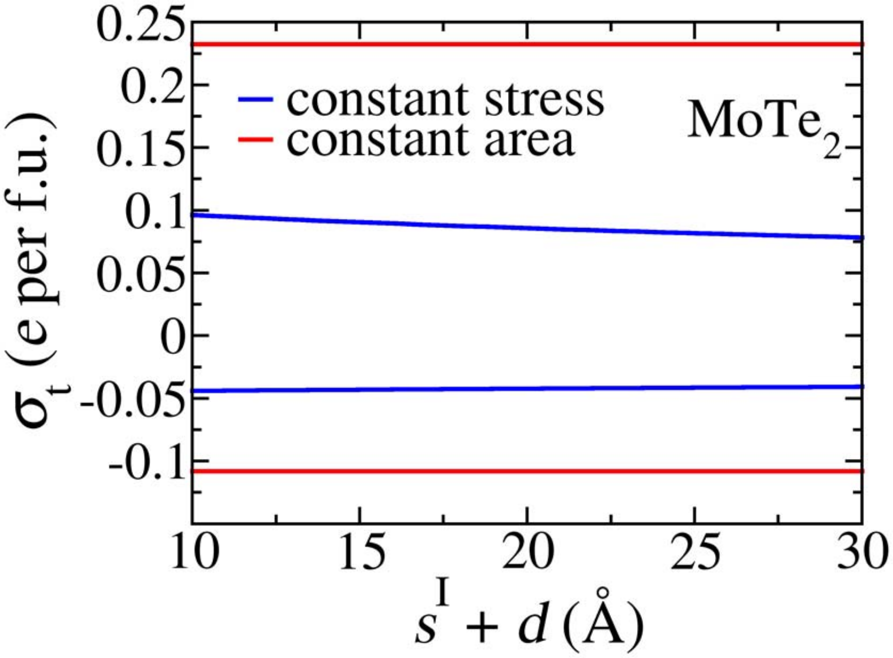

Supplementary Figure 7: Dependence of transition charge densities on distance parameter. Plotted are transition charge densities with respect to the distance between $MoTe_2$ center and the surface of an electron reservoir $s^I + d$ (shown in Fig. 3 of main text). The transition charge densities have a small dependence on the distance $s^I + d$ in constant-stress case (blue lines), and no dependence on the distance in constant-area case (red lines).

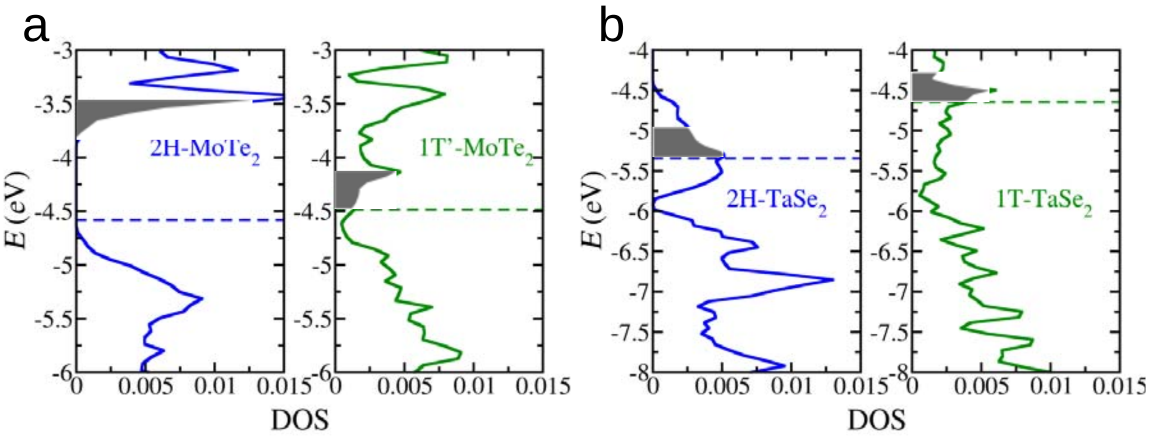

Supplementary Figure 8: Electronic density of Kohn-Sham states for monolayer $MoTe_2$ (a) and $TaSe_2$ (b). Kohn-Sham energies (y axis) are shifted so that zero corresponds to the vacuum level in each case. The dashed lines label the Fermi level positions. Semiconducting 2H-$MoTe_2$ has a band gap of 0.95 *e*V, and 1T'-$MoTe_2$ has no band gap. Both 2H- and 1T'-$TaSe_2$ are metallic without band gap. The shaded regions depict the additional states occupied in negatively-charged monolayer.

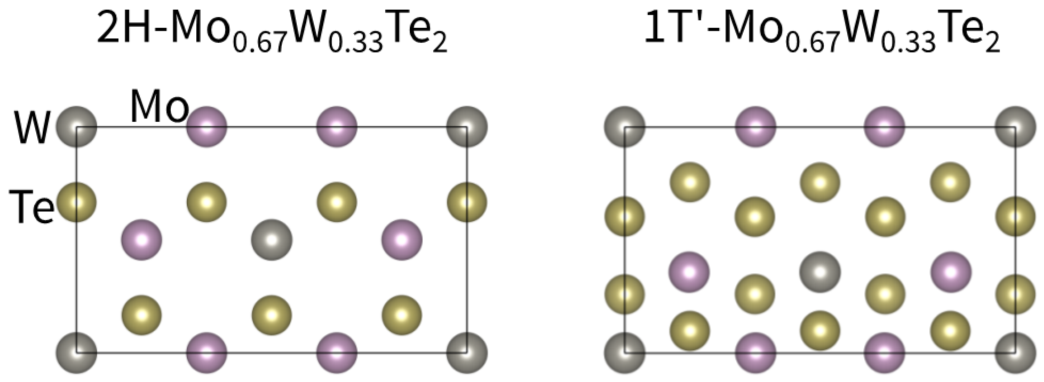

Supplementary Figure 9: Computational cells of the crystalline phases of alloy $Mo_{0.67}W_{0.33}Te_2$ employed in this work. Plotted are top views of computational cells of 2H and 1T' phases of a representative alloyed monolayer $Mo_{0.67}W_{0.33}Te_2$. Each computational cell consists of 12 Tellurium atoms (shown in yellow), 4 Molybdenum atoms (shown in purple), and 2 Tungsten atoms (shown in grey). The k-points are sampled on a $16 \times 16 \times 1$ Monkhorst-Pack[2] grid. A vacuum space of 36 Å is used along the z direction.

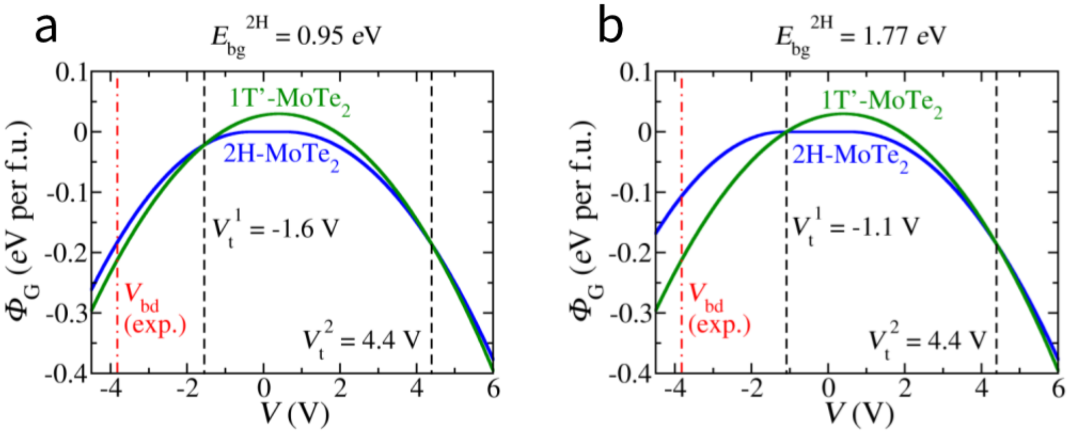

Supplementary Figure 10: The effect of band gap on the phase boundary at constant voltage. Panel **a** is the same as Fig. 4(c) in main text, where the PBE-DFT band gap of 0.95 eV is used for 2H-$MoTe_2$. Panel **b** is similar to panel **a**, except that the band gap of 2H-MoTe2 is increased to 1.77 eV by shifting the conduction band. The positive transition voltage V_t^2 does not change, but the negative transition voltage V_t^1 shifts to the right and the magnitude is reduced from 1.6 V to 1.1 V.

Supplementary Table 1: Distance parameters in Å for bulk 2H-$MoTe_2$, 2H-MoS_2, and 2H-$TaSe_2$. The meaning of each parameter is labeled in Supplementary Fig. 5.

Distance Parameter	2H-$MoTe_2$ (Å)	2H-MoS_2 (Å)	2H-$TaSe_2$ (Å)
c	6.98[3]	6.15[3]	6.37[4]
t	1.80[3]	1.59[3]	1.67[*]
r	1.40[5]	1.00[5]	1.15[5]
$t + r$	3.20	2.59	2.82
$s_0 = c - t - r$	3.78	3.56	3.55

[*] Calculated using PBE-DFT in this work.

Supplementary Note 1: Energy calculations of charged monolayers using DFT.

In a DFT simulation of a charged monolayer with periodic boundary conditions using Vienna Ab Initio Simulation Package (VASP)[6], a homogeneous background charge is automatically introduced in the vacuum space in order to compensate for the excess charge, allowing for the periodic computational cell to remain electrically neutral and the total electrostatic energy to remain finite. A reference plane parallel to the monolayer is chosen, at a distance z_{ref} away from the monolayer center, as shown in Supplementary Fig. 1. The full energy of the charged monolayer $E^{\mathrm{mo}}(Q,\ z_{\mathrm{ref}})$ is calculated from

$$E^{\mathrm{mo}}(Q,\ z_{\mathrm{ref}}) = E_0 + \int_0^Q \Delta V(Q',\ z_{\mathrm{ref}})dQ' \qquad (1)$$

where E_0 is the ground-state energy of an electrically neutral monolayer, the integral is the energy of moving charge Q from the reference plane to the Fermi level of the monolayer, and $\Delta V(Q',\ z_{\mathrm{ref}})$ is the electronic potential difference between the reference plane z_{ref} and the surface of the material defined as z_{f}. We take z_{f} to be the plane at which the plane-averaged Kohn-Sham potential is equal to Fermi level, as shown in Supplementary Fig. 1. This position changes with the charge in the monolayer, i.e. $z_{\mathrm{f}} = z_{\mathrm{f}}(Q')$, defined via:

$$V_{\mathrm{tot}}\big(Q',\ z_{\mathrm{f}}(Q')\big) = \mu_{\mathrm{f}}(Q') \qquad (2)$$

The potential difference $\Delta V(Q',\ z_{\mathrm{ref}})$ in equation (1) is,

$$\Delta V(Q',\ z_{\mathrm{ref}}) = [V_{\mathrm{tot}}(Q',\ z_{\mathrm{ref}}) - \mu_{\mathrm{f}}(Q')] - \big[V_{\mathrm{bg}}(Q',\ z_{\mathrm{ref}}) - V_{\mathrm{bg}}\big(Q', z_{\mathrm{f}}(Q')\big)\big] + \frac{Q'}{2\varepsilon_0 A}[z_{\mathrm{ref}} - z_{\mathrm{f}}(Q')] \qquad (3)$$

where V_{bg} is the electrostatic potential generated by the uniform compensating background charge $-Q'$ and A is the area of the monolayer.

The background potential V_{bg} in equation (3) is given by

$$V_{\mathrm{bg}}(Q',\ z) = \frac{Q\prime}{2\varepsilon_0 AL}\left(z^2 - \frac{1}{4}L^2\right) \qquad (4)$$

where L is the size of the computational cell along *c,* interlayer axis. Equation (4) assumes that the monolayer is placed at $z = 0$.

The third and fourth terms in equation (3) are included to subtract the effect of the compensating background charge. Supplementary Fig. 1 shows that after subtracting the background charge effect, a uniform electric field remains on both sides in the vacuum caused by a charged monolayer, as is the standard electrostatic result for a charged plane. The magnitude of the uniform electric field on both sides can be computed using Gauss's law, $\frac{Q'}{2\varepsilon_0 A}$. This magnitude is only half of the magnitude of the electric field inside a parallel capacitor with charge Q' on each plate. Therefore, the last two terms in equation (3) are added to restore the magnitude of the electric field in a parallel capacitor consisting of a monolayer charged with Q'.

The process of obtaining an analytical expression for the energy of a charged monolayer $E^{\mathrm{mo}}(Q,\ z_{\mathrm{ref}})$ in equation (1) can be described as follows:

First, DFT simulations are performed for a monolayer charged with different values of Q'. For monolayer TMDs, Q' is chosen to vary from -0.06 to 0.15 e per formula unit with an increment of 0.01 e per formula unit, and a vacuum space of 36 Å is used in all cases. Secondly, a reference plane $z_0 = z_{\mathrm{ref}}$ is chosen. For monolayer TMDs, this distance is chosen to be 17.25 Å. Thirdly, for each value of Q', the potential difference between the reference plane at z_0 and the surface of the material $\Delta V(Q',\ z_0)$ can be calculated using equation (3). Fourth, first-degree polynomial fitting is performed using a linear least squares regression method to get an analytical expression for $\Delta V(Q',\ z_0)$ as a function of Q', which can be written as:

$$\Delta V(Q',\ z_0) = a_1 + 2{a_2}'Q' \qquad (5)$$

where a_1 and ${a_2}'$ are fitting coefficients. Supplementary Fig. 1 shows that at a large value of $z_0 = z_{\mathrm{ref}} = 17.25$ Å, the potential difference between z_{ref} and z_{f} (blue dashed line) is primarily determined by the uniform electric field, which is in direct proportion to Q'. Therefore, linear fitting is an appropriate first-order approximation. Supplementary Fig. 2 shows the fitting results of Equation (5) for 2H- and 1T'-$MoTe_2$, and the root-mean-squared error is smaller than 0.08 V. The discontinuity at $Q' = 0$ in semiconducting 2H-$MoTe_2$ is due to the band gap.

Fifth, the potential difference at any other reference plane z_{ref}, $\Delta V(Q',\ z_{\mathrm{ref}})$, can be computed from equation (5) using the uniform electric field:

$$\Delta V(Q',\ z_{\mathrm{ref}}) = \Delta V(Q', z_0) + \frac{Q'}{\varepsilon_0 A}(z_{\mathrm{ref}} - z_0) = a_1 + \left(\frac{z_{\mathrm{ref}}}{\varepsilon_0 A} + 2a_2' - \frac{z_0}{\varepsilon_0 A}\right)Q' = a_1 + \left(\frac{z_{\mathrm{ref}}}{\varepsilon_0 A} + 2a_2\right)Q' \qquad (6)$$

where $a_2 = a_2' - \frac{z_0}{2\varepsilon_0 A}$.

Last, the full energy of the charged monolayer, $E^{\mathrm{mo}}(Q,\ z_{\mathrm{ref}})$, can be calculated using equation (1) and (6):

$$E^{\mathrm{mo}}(Q,\ z_{\mathrm{ref}}) = E_0 + a_1 Q + \left(\frac{z_{\mathrm{ref}}}{2\varepsilon_0 A} + a_2\right) Q^2 \qquad (7)$$

Supplementary Note 2: Test case of a monolayer $MoTe_2$ on top of a Cs substrate.

In the main text, we have discussed how to calculate the energy of a system consisting of a charged monolayer, and in Supplementary Note 1 we have discussed energy calculations of charged monolayers in DFT with periodic boundary conditions. To test and obtain confidence intervals for the approach we have proposed, we have simulated a system consisting of a monolayer $MoTe_2$ placed on a Cs substrate. The metal substrate is chosen to be Cs because Cs has a low work function to facilitate the transfer of charge from the substrate to the monolayer $MoTe_2$.

Each computational cell includes 4 formula units of $MoTe_2$ and 4 or 6 Cs atoms, with a cell size of 60 Å in the direction perpendicular to the monolayer. Supplementary Fig. 3, panel a shows a top view of the computational cell and a side view of the computational cell replicated in the x and y directions. The computational cell parameters are constrained to the relaxed lattice parameters of isolated $MoTe_2$, and the Cs substrate (bcc structure) is strained to fit into this computational cell. The lattice constant in the *z* direction for the Cs substrate is tuned so that the substrates for computations with the

2H and 1T’ phases have the same work function of 1.9 eV. In these simulations, atom coordinates are fixed and only electronic optimization is performed.

To compute the energy difference between phases, we first define some notation. Let the energy of the Cs substrate computed at the H lattice constants $E(Cs^H)$, and the total energy of the system consisting of 2H-$MoTe_2$ on Cs substrate is E(2H-$MoTe_2$ + Cs^H). We adopt similar notation for 1T’-$MoTe_2$. Because the Cs substrate is strained to fit into the computational cell, it has nonzero strain energy, which is defined as the energy difference between the strained Cs substrate and a zero-stress Cs substrate. The energy of a zero-stress Cs substrate is denoted as $E(Cs^0)$. We take the energy difference between a system consisting of 2H-$MoTe_2$ and a system consisting of 1T’-$MoTe_2$ ΔE^{simul} as,

$$\Delta E^{\mathrm{simul}} = \left\{E\left(1\mathrm{T}' - \mathrm{MoTe_2} + \mathrm{Cs}^{\mathrm{T}'}\right) - \left[E\left(\mathrm{Cs}^{\mathrm{T}'}\right) - E(\mathrm{Cs}^0)\right]\right\}$$
$$- \{E(2\mathrm{H} - \mathrm{MoTe_2} + \mathrm{Cs}^{\mathrm{H}}) - [E(\mathrm{Cs}^{\mathrm{H}}) - E(\mathrm{Cs}^0)]\}$$
$$= [E(1\mathrm{T}' - \mathrm{MoTe_2} + \mathrm{Cs}^{\mathrm{T}'}) - E(2\mathrm{H} - \mathrm{MoTe_2} + \mathrm{Cs}^{\mathrm{H}})] - [E(\mathrm{Cs}^{\mathrm{T}'}) - E(\mathrm{Cs}^{\mathrm{H}})], \quad (8)$$

where the strain energy of the Cs substrate has been subtracted.

Plotted in Supplementary Fig. 3, panel b is the averaged electrostatic potential along the vacuum direction obtained from DFT simulation. A uniform electric field E_{field} between the monolayer and the substrate is generated by the charge Q transferred between them,

$$E_{\text{field}} = \frac{Q}{\varepsilon_0 A}, \quad (9)$$

where A is monolayer area.

Using the approach we have proposed to make model predictions for such a system (Supplementary Note 1), we first perform separate DFT calculations for isolated monolayer $MoTe_2$ and isolated Cs substrates. The energy of a $MoTe_2$ monolayer $E^{\text{mo}}(Q,\ s^{\text{I}})$ and the energy of a Cs substrate $E^{\text{sub}}(-Q,\ s^{\text{II}})$ can be computed using equation (7):

$$E^{\text{mo}}(Q,\ s^{\text{I}}) = E_0{}^{\text{mo}} + a_1{}^{\text{mo}} Q + \left(\frac{s^{\text{I}}}{2\varepsilon_0 A} + a_2{}^{\text{mo}}\right) Q^2 \quad (10)$$

$$E^{\text{sub}}(-Q,\ s^{\text{II}}) = E_0{}^{\text{sub}} - a_1{}^{\text{sub}} Q + \left(\frac{s^{\text{II}}}{2\varepsilon_0 A} + a_2{}^{\text{sub}}\right) Q^2 \quad (11)$$

where s^{I} is the distance from the center of $MoTe_2$ to the surface of the uniform electric field region and s^{II} is the distance from the surface Cs atom to the other surface of the uniform electric field region, as shown in Fig. 2 of main text. Therefore, the separation between the center of $MoTe_2$ and the surface Cs atoms can be written as

$$d_{\text{Mo-Cs}} = s^{\text{I}} + s^{\text{II}} + d \quad (12)$$

The total energy of the system $E(Q, d_{\text{Mo-Cs}})$ can be computed using Equation (1) of the main text:

$$E(Q, d_{\text{Mo-Cs}}) = E^{\text{mo}}(Q,\ s^{\text{I}}) + E^{\text{sub}}(-Q,\ s^{\text{II}}) + E_{\text{c}} \quad (13)$$

Using equations (10) and (11), we can further compute:

$$E(Q, d_{\mathrm{Mo-Cs}}) = {E_0}^{\mathrm{mo}} + {a_1}^{\mathrm{mo}} Q + \left(\frac{s^{\mathrm{I}}}{2\varepsilon_0 A} + {a_2}^{\mathrm{mo}}\right) Q^2 + {E_0}^{\mathrm{sub}} - {a_1}^{\mathrm{sub}} Q + \left(\frac{s^{\mathrm{II}}}{2\varepsilon_0 A} + {a_2}^{\mathrm{sub}}\right) Q^2 + \frac{d}{2\varepsilon_0 A} Q^2 = {E_0}^{\mathrm{mo}} + {E_0}^{\mathrm{sub}} + \left({a_1}^{\mathrm{mo}} - {a_1}^{\mathrm{sub}}\right) Q + \left(\frac{d_{\mathrm{Mo-Cs}}}{2\varepsilon_0 A} + {a_2}^{\mathrm{mo}} + {a_2}^{\mathrm{sub}}\right) Q^2 \qquad (14)$$

As discussed in the main text, the equilibrium charge transferred, Q_{eq}, can be computed through minimization of the total energy $E(Q, d_{\mathrm{Mo-Cs}})$ (equivalent to the grand potential since no gate voltage is applied). After which, we can further compute the total energy at equilibrium:

$$E^{\mathrm{eq}}(d_{\mathrm{Mo-Cs}}) = E\left(Q_{\mathrm{eq}}, d_{\mathrm{Mo-Cs}}\right) \qquad (15)$$

The model prediction results from equation (14) and DFT simulation results are compared in Supplementary Fig. 3, panels c and d. Plotted in Supplementary Fig. 3(c) is the difference between the simulation and model prediction for the charge transferred from the substrate to 2H-$MoTe_2$ as a function of $d_{\mathrm{Mo-Cs}}$. Plotted in Supplementary Fig. 3(d) is the voltage difference between the simulation and model prediction, estimated by dividing the difference between the simulation and model prediction for the energy difference of a system consisting of 2H-$MoTe_2$ and a system consisting of 1T'-$MoTe_2$ by the excess charge in 2H-$MoTe_2$. For the blue curves, the Cs substrate consists of 4 layers; for the red curves, the Cs substrate consists of 6 layers. Consistency between the 4-Cs-

substrate-layer case (blue curves) and 6-Cs-substrate-layer case (red curves) shows that the number of Cs layers is sufficient to adequately describe the bulk electrode.

Supplementary Fig. 3 panels c and d show that the differences between the DFT simulation and the model prediction are converged at the separation $d_{\mathrm{Mo-Cs}}$ larger than 10 Å. The separation $d_{\mathrm{Mo-Cs}} = d + s^{\mathrm{I}} + s^{\mathrm{II}}$, the distance between the center of the monolayer TMDs and the electron reservoir, is chosen to be larger than 10 Å in this work. Therefore the charge density difference is converged to approximately 0.005 e per formula unit and the voltage difference is converged to approximately -0.1 V for the results presented here. Sources for this error may include the first-order approximation employed in the fitting of $\Delta V(Q',\ z_0)$, as shown in equation (5).

Supplementary Note 3: Distance parameters of TMDs.

The distance between a monolayer TMDs and the substrate, s^I, may vary over a few Angstroms[10,11]. DFT-based calculations of the average separation between the Mo layer of monolayer MoS_2 and the top layer of substrate has been reported to vary, e.g. from 3.57 Å in the case of Ti-substrate to 4.21 Å in the case of Au-substrate[12]. When identifying appropriate parameters for monolayer TMDs, one can refer to distance parameters in bulk TMDs. Some distance parameters in bulk TMDs in the 2H phase are labeled in Supplementary Fig. 5. Parameter c is the distance between the centers of two neighboring layers. Parameter t is the distance from the transition metal atom centers to the chalcogenide atom centers. Parameter s_0 is the distance from the transition

metal atom centers of one layer to the chalcogenide atom surfaces of the neighboring layer. Parameter r represents the distance from the chalcogenide atom centers to the chalcogenide atom surfaces, and is estimated from empirically determined atomic radii[5]. The values used for these parameters are listed in Supplementary Table 1. The parameters for 1T'/1T phases are taken to be the same as those for 2H phase. For the capacitor structure shown in Fig. 4(a) in main text, s is chosen to be $s_0 = c - t - r$ (see values in Supplementary Table 1).

The dependence of transition voltages on the separation s is explored in Supplementary Fig. 6 ($s = s^{\mathrm{I}}$). Supplementary Fig. 6 shows that the magnitudes of transition voltages increase modestly with the separation between monolayer TMDs and substrate. The phase boundaries vary in position with the distance s between $MoTe_2$ center and the surface of dielectric medium by 0.4 V for negative charging and 1.0 V for positive charging over a physical range of s.

Supplementary Note 4: Thermodynamic potentials in different conditions.

All DFT calculations are performed at 0 K ionic and electronic temperature.

$$T = 0 \qquad (16)$$

In the stress-free case, both 2H and 1T' phases are structurally relaxed at a condition of constant in-plane stress, and $P = 0$. Therefore, Gibbs free energy G is the relevant potential, and is written as:

$$G = U + PV - TS = U \qquad (17)$$

where U is internal energy.

In the scenario of constant area, 1T' is constrained to the computational cell of the relaxed 2H phase. Therefore, Helmholtz free energy F should be computed, and is written as:

$$F = U - TS = U \qquad (18)$$

Because free energy is the same as internal energy in either case, they are denoted with the same symbol E in main text, although it represents different thermodynamic potentials in different conditions as discussed here.

Supplementary Note 5: Mechanism for charge-induced structural phase transition.

Despite the earlier theoretical studies on structural phase transitions in monolayer TMDs driven by strain[1,7] and heating[8], the mechanism for such a structural phase transition induced by charge is not understood. Here, we explain the mechanism for such a charge-induced phase transition using energy band diagrams[9]. Supplementary Fig. 8 shows the electronic Kohn-Sham density of states for monolayer $MoTe_2$ and $TaSe_2$ with Kohn-Sham energies (y axis) shifted so that zero corresponds to the vacuum level in each case. 2H phase has a lower free energy when the monolayer is electrically neutral in both cases. Supplementary Fig. 8(a) shows that semiconducting 2H-$MoTe_2$ has a Kohn-Sham band gap while 1T'-phase has no band gap. When monolayer $MoTe_2$ is negatively charged, the excess electrons will occupy the lowest energy states above the

Fermi energy (shaded regions), which are 0.8 eV higher for 2H than for 1T' $MoTe_2$ in this case. The large band gap in the 2H-phase makes the electronic energy of sufficiently negatively charged monolayers higher in the 2H-$MoTe_2$ phase than in the 1T'-$MoTe_2$ phase. A similar phenomenon occurs in the case of positive charging, when electrons are removed below the Fermi levels. The Fermi level of 1T'-$MoTe_2$ is higher than the valence band of the 2H phase; therefore it requires more energy to take electrons out of 2H-$MoTe_2$ than the 1T' phase, resulting in a lower 1T' phase energy for sufficiently positive charge. The presence of a gap in the 2H phase leads to a phase transition in $MoTe_2$ for both positive and negative charge.

Supplementary Fig. 8(b) shows that the case of $TaSe_2$ is a metal-to-metal transition which gives rise to a phase boundary at only one sign of the charge. When $TaSe_2$ is sufficiently positively charged, the 1T phase will be favored because the Fermi level in 1T-$TaSe_2$ is higher than that in the 2H phase. However, in contrast to $MoTe_2$, 2H-$TaSe_2$ always has a lower free energy than the 1T phase when the monolayer is negatively charged.

The simplistic picture presented in Supplementary Fig. 8 suggests that other monolayers that exhibit metal-metal transitions are most likely to exhibit a phase boundary at one sign (positive or negative) while a gap in one of the phases can lead to transitions at both positive and negative charges.

Note that this charging mechanism is distinct from the application of an electric field to an electrically isolated monolayer, where the charge on the monolayer will remain neutral. Charge must be allowed to flow on and off the monolayer to achieve the transition described in this work.

Supplementary Note 6: The effect of band gap width.

The quasiparticle Kohn-Sham band gap of 2H-$MoTe_2$ calculated using the PBE XC functional is 0.95 *e*V, smaller than the optical band gap measured in experiment (1.10 eV)[13] and the quasiparticle band gap reported using a GW correction (1.77 eV)[14]. In Supplementary Fig. 10, panel a (the same as Fig. 3(c) in main text), the top of the curve for semiconducting 2H-$MoTe_2$ is observed as a plateau of width equivalent to the band gap width. To estimate the effect of band gap width on transition voltage, we increase the band gap to 1.77 *e*V by shifting the conduction band while fixing the valence band, and the result is plotted in Supplementary Fig. 10, panel b. As shown in Supplementary Fig. 10, the positive transition voltage ${V_t}^2$ does not change, which is expected since it is mainly determined by the valence band. The negative transition voltage ${V_t}^1$, however, is mainly determined by the conduction band, and its magnitude is reduced from 1.6 V to 1.1 V. It is known that the PBE functional usually underestimates band gaps. This may result in an over-estimation of the magnitude of the negative transition gate voltage for the group VI TMDs in this work.

Supplementary Note 7: Vacuum electronic states.

When excess electrons are assigned to the computational cell, a homogeneous positive background charge is automatically introduced in the vacuum space in order to compensate the excess charge. When the number of excess electrons is increased to some value and the vacuum separation in the direction perpendicular to the monolayer surface is bigger than some corresponding threshold, the Kohn-Sham states will begin to occupy low-lying vacuum electronic states in the center of the vacuum region, at the boundary of the computational cell. Special attention has been given to avoid the formation of these vacuum electronic states by not adding too many excess electrons.

Supplementary References

1. Duerloo, K.-A. N., Li, Y. & Reed, E. J. Structural phase transitions in two-dimensional Mo- and W-dichalcogenide monolayers. *Nat. Commun.* **5,** (2014).
2. Monkhorst, H. J. & Pack, J. D. Special points for Brillouin-zone integrations. *Phys. Rev. B* **13,** 5188–5192 (1976).
3. Böker, T. *et al.* Band structure of MoS2, MoSe2, and α-MoTe2: Angle-resolved photoelectron spectroscopy and ab initio calculations. *Phys. Rev. B* **64,** 235305 (2001).
4. Bjerkelund, E. *et al.* On the Structural Properties of the Ta(1+x)Se2 Phase. *Acta Chem. Scand.* **21,** 513–526 (1967).
5. Slater, J. C. Atomic Radii in Crystals. *J. Chem. Phys.* **41,** 3199–3204 (1964).

6. Kresse, G. & Furthmüller, J. Efficient iterative schemes for ab initio total-energy calculations using a plane-wave basis set. *Phys. Rev. B* **54,** 11169–11186 (1996).
7. Kan, M., Wang, B., Lee, Y. H. & Sun, Q. A density functional theory study of the tunable structure, magnetism and metal-insulator phase transition in VS2 monolayers induced by in-plane biaxial strain. *Nano Res.* **8,** 1348–1356 (2014).
8. Duerloo, K.-A. N. & Reed, E. J. Structural Phase Transitions by Design in Monolayer Alloys. *ACS Nano* (2015). doi:10.1021/acsnano.5b04359
9. Py, M. A. & Haering, R. R. Structural destabilization induced by lithium intercalation in MoS2 and related compounds. *Can. J. Phys.* **61,** 76–84 (1983).
10. Gutiérrez, H. R. *et al.* Extraordinary Room-Temperature Photoluminescence in Triangular WS2 Monolayers. *Nano Lett.* **13,** 3447–3454 (2013).
11. Gong, C. *et al.* Metal Contacts on Physical Vapor Deposited Monolayer MoS2. *ACS Nano* **7,** 11350–11357 (2013).
12. Popov, I., Seifert, G. & Tománek, D. Designing Electrical Contacts to MoS2 Monolayers: A Computational Study. *Phys. Rev. Lett.* **108,** 156802 (2012).
13. Ruppert, C., Aslan, O. B. & Heinz, T. F. Optical Properties and Band Gap of Single- and Few-Layer MoTe2 Crystals. *Nano Lett.* **14,** 6231–6236 (2014).
14. Ding, Y. *et al.* First principles study of structural, vibrational and electronic properties of graphene-like MX2 (M=Mo, Nb, W, Ta; X=S, Se, Te) monolayers. *Phys. B Condens. Matter* **406,** 2254–2260 (2011).
